# The World Federation of Chiropractic Global Patient Safety Task Force: a call to action

**DOI:** 10.1186/s12998-024-00536-1

**Published:** 2024-05-13

**Authors:** Brian C. Coleman, Sidney M. Rubinstein, Stacie A. Salsbury, Michael Swain, Richard Brown, Katherine A. Pohlman

**Affiliations:** 1 Department of Emergency Medicine, Yale School of Medicine, New Haven, CT USA; 2 Department of Biostatistics (Health Informatics), Yale School of Public Health, New Haven, CT USA; 3 Pain Research, Informatics, Multimorbidities, and Education (PRIME) Center, VA Connecticut Healthcare System, West Haven, CT USA; 4 Department of Health Sciences, Faculty of Science, Amsterdam Movement Sciences Research Institute, Vrije Universiteit Amsterdam, Amsterdam, the Netherlands; 5 Palmer Center for Chiropractic Research, Palmer College of Chiropractic, Davenport, IA USA; 6 Department of Chiropractic, Macquarie University, Sydney, Australia; 7 World Federation of Chiropractic, Toronto, Canada; 8 Research Center, Parker University, 2540 Walnut Hill Lane, 75229 Dallas, TX USA

**Keywords:** Patient safety, Chiropractic, Safety management, Risk management, World Health Organization, Medical errors, Patient harm, Health personnel

## Abstract

**Background:**

The Global Patient Safety Action Plan, an initiative of the World Health Organization (WHO), draws attention to patient safety as being an issue of utmost importance in healthcare. In response, the World Federation of Chiropractic (WFC) has established a Global Patient Safety Task Force to advance a patient safety culture across all facets of the chiropractic profession. This commentary aims to introduce principles and call upon the chiropractic profession to actively engage with the Global Patient Safety Action Plan beginning immediately and over the coming decade.

**Main text:**

This commentary addresses why the chiropractic profession should pay attention to the WHO Global Patient Safety Action Plan, and what actions the chiropractic profession should take to advance these objectives. Each strategic objective identified by WHO serves as a focal point for reflection and action. Objective 1 emphasizes the need to view each clinical interaction as a chance to improve patient safety through learning. Objective 2 urges the implementation of frameworks that dismantle systemic obstacles, minimizing human errors and strengthening patient safety procedures. Objective 3 supports the optimization of clinical process safety. Objective 4 recognizes the need for patient and family engagement. Objective 5 describes the need for integrated patient safety competencies in training programs. Objective 6 explains the need for foundational data infrastructure, ecosystem, and culture. Objective 7 emphasizes that patient safety is optimized when healthcare professionals cultivate synergy and partnerships.

**Conclusions:**

The WFC Global Patient Safety Task Force provides a structured framework for aligning essential considerations for patient safety in chiropractic care with WHO strategic objectives. Embracing the prescribed action steps offers a roadmap for the chiropractic profession to nurture an inclusive and dedicated culture, placing patient safety at its core. This commentary advocates for a concerted effort within the chiropractic community to commit to and implement these principles for the collective advancement of patient safety.

**Supplementary Information:**

The online version contains supplementary material available at 10.1186/s12998-024-00536-1.

## Background

Patient safety is paramount across healthcare to prevent avoidable harms, support evidence-based clinical care, and maintain public trust [1, 2]. In 2023, the World Federation of Chiropractic (WFC) Board of Directors unanimously approved the formation of a Global Patient Safety Task Force to cultivate patient safety culture across the chiropractic profession. This task force aims to support chiropractors worldwide in prioritizing patient safety in their professional activities, including sharing best practices, dispelling misinformation, enhancing treatment safety, improving interprofessional collaboration, and supporting public health. Towards this mission, we urge the chiropractic profession to adopt a call to action informed by the *World Health Organization (WHO) Global Patient Safety Action Plan* to envision, implement, and advance a robust patient safety culture in the coming decade.

### Call to action

The *WHO Global Patient Safety Action Plan* creates the vision of “a world in which no one is harmed in health care and every patient receives safe and respectful care, every time, everywhere” [1]. Global partnerships among all domains of healthcare, including chiropractic, must take precedence to achieve this shared vision [3]. This call to action encourages chiropractic stakeholders to commit to patient safety, reduce avoidable harm for chiropractic patients, and position the profession as an exemplar of safety culture among healthcare professions. This necessitates adoption of the *WHO Global Patient Safety Action Plan* to identify areas of excellence and create opportunities to improve patient safety across professional organizations, health systems and clinics, and educational settings.

### A framework to promote patient safety in chiropractic healthcare

**Fig. 1:**
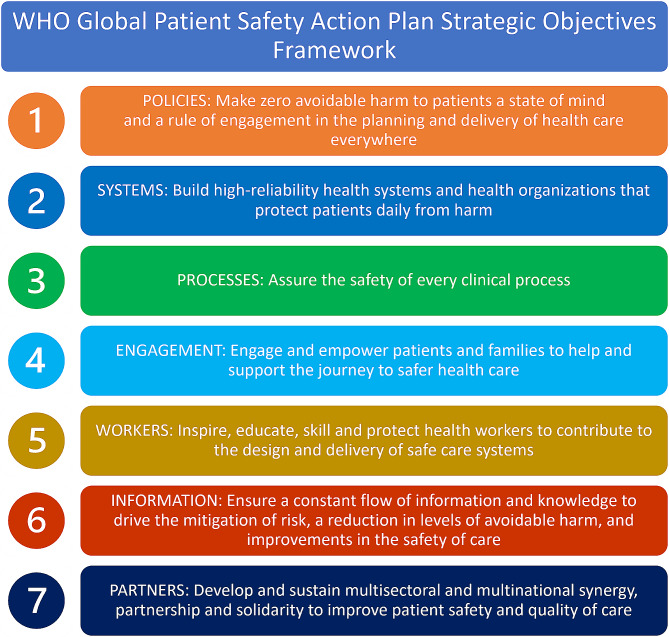
Strategic objectives of the WHO Global Patient Safety Action Plan (Adapted from Global patient safety action plan 2021–2030: towards eliminating avoidable harm in healthcare. Geneva: World Health Organization; 2021. License: CC BY-NC-SA 3.0 IGO)

The *WHO Global Patient Safety Action Plan* provides a practical framework to guide knowledge translation, program planning and implementation, and outcome evaluation for patient safety initiatives [1]. Seven strategic objectives address the interrelated building blocks of patient safety including policies, systems, processes, patient and family engagement, healthcare workers, information, and partnerships (Fig. 1). In this commentary, we contextualize each objective with considerations and action items to address: (1) why the chiropractic profession should prioritize the WHO Global Patient Safety Action Plan, and (2) what actions can, and should, the chiropractic profession takes to advance these objectives. The seven strategic objectives, with considerations and action items, are outlined below. Expanded considerations and actions are available in a supplemental table.

#### Objective 1. Policies to eliminate avoidable harm in chiropractic

The first *WHO Global Patient Safety Action Plan* strategic objective holds health organizations accountable for patient safety. While intuitive, healthcare is less responsive than other industries, such as aviation, in maintaining environments that prevent safety events despite a *prima facie* responsibility to avoid patient harm [2, 4, 5]. Healthcare, including chiropractic, requires a paradigm shift to embrace a patient safety culture that leads to positive patient outcomes and a reduction in avoidable harm [6]. 

### Considerations

Healthcare workers and organizations exhibit varied interest and engagement in patient safety activities [7]. Health professionals have been shown to be demotivated to focus on patient safety when the risk of harm is mild and transient, as is the case with most common adverse events associated with chiropractic care [8]. Broader awareness of the multifaceted nature of patient safety beyond adverse events reveals numerous opportunities to improve patient safety [5]. Examples include policies and practices to improve informed consent, health equity, interprofessional communication, facilities management, and more.

### Call to action

Chiropractic professional organizations must adopt equitable policies affirming a commitment to eliminating avoidable harm in healthcare as a health priority. Regulatory and clinic policies should be reviewed and updated periodically to ensure professional standards in licensing, continuing education, accreditation, and other regulatory domains affirm patient safety culture across jurisdictions. Sufficient resources to support policy enactment, including financial and human resources, must be allocated to achieve patient safety goals. Policy development should include advancing a research agenda for patient safety in chiropractic.

#### Objective 2. High-reliability safety systems

The second strategic objective of the *WHO Global Patient Safety Action Plan* develops high-reliability systems to support patient safety in healthcare organizations. Inspiration may be drawn from high-risk industries, such as aviation and nuclear power, who establish high-reliability systems to proactively anticipate and address safety issues rather than relying on individuals to prevent mistakes or solely reacting to adverse events [2, 5, 9]. Resilient systems focused on both learning from failure and facilitating success that align with an established framework, that is: Safety-I, which presumes things go wrong because of identifiable failures or malfunctions of specific components; and Safety-II, which presumes humans are resources that adapt to varying conditions and they are the reason why things go right [5]. 

### Considerations

Most health professions, including chiropractic, focus on the Safety-I Framework to minimize serious but rare adverse outcomes. The Safety-II Framework recognizes healthcare delivery as typically uneventful with respect to patient safety. Safety-II encourages stakeholders to approach safety with openness, transparency, and constant learning to address human and workplace factors that prevent optimal safety environments. These frameworks together can build a just patient safety culture that recognizes complexities in healthcare environments.

### Call to action

The chiropractic profession must commit to resilient, high-reliability systems by adopting the Safety-I and Safety-II Frameworks. Organizations should conduct regular reporting, surveillance, and critical appraisal of both rare and ordinary safety concerns to understand what creates success and where gaps exist in their patient safety culture.

#### Objective 3. Safety of clinical processes

The *WHO Global Patient Safety Action Plan’s* third strategic objective focuses on overcoming complex clinical care challenges created by unclear processes and interconnected procedures occurring across vast healthcare systems. Nearly all patient safety incidents stem from flaws within these clinical operations systems [2, 10]. Resilient design factors must be tailored to the circumstances of each clinical setting, including environmental and physical factors, team factors, organizational culture and structure, and regulation and policy.

### Considerations

Poor communication between providers, patients, and facilities can undermine the safety of clinical processes leading to errors of omission, misdiagnosis, or failure-to-monitor incidents [11]. Qualitative studies identify a need for improved communication by community-based chiropractors and among faculty and students in chiropractic teaching clinics [3, 12]. 

### Call to action

Optimal communication processes between chiropractors and the broader healthcare system needs to be prioritized to enhance patient safety. Standard operating procedures must be used consistently in each healthcare event to improve diagnosis, treatment, referral, and follow-up for chiropractic patients. Standardized assessment tools that identify chiropractic patients at risk for safety events should be developed and used to improve clinical process safety.

Standardized clinical processes must be structured to assist clinicians in ruling out “red-flags”. This will increase the likelihood of identifying patients presenting to the chiropractor with serious and potentially-life threatening conditions needing immediate action, for example, headache or neck pain symptoms with a high suspicion of a cerebrovascular accident in-progress, or low back pain symptoms consistent with a cauda equina syndrome [13]. 

#### Objective 4. Patient and family engagement

The fourth *WHO Global Patient Safety Action Plan* strategic objective centers on patient and family empowerment. The person at the center of a healthcare event– the patient– must be an essential partner in safety initiatives. WHO advocates for an engagement paradigm in which patients, families, and caregivers are respected as equal partners in patient safety. Problematically, few studies have incorporated the critical insights and perspectives of patients and families regarding patient safety [14–19]. Learning from patients’ experiences of healthcare will require healthcare leaders to engage in systematic activity to welcome patients and families with diverse backgrounds onto safety committees [20]. 

### Considerations

Patient and family advocates should be valued, supported, and meaningfully engaged in advancing cultures of safety and respect in chiropractic healthcare settings. This includes incorporating patients and family members as partners in policy development and quality improvement initiatives targeting safety, championing their experience and providing a voice. In the chiropractic setting, ensuring bilateral commitment to the informed consent process with full and transparent disclosure of potential risks is one example of meaningful engagement.

### Call to action

The chiropractic profession must sustain patient-centered environments [21–24] aligned with professional principles committing to ethical and safe care [25]. Training programs on how to engage in constructive dialogue with patients, including exercising the duty of candor and instruction in how to respond to safety events when they occur, need to be encouraged. Appointing patients to representative and advisory roles on safety initiatives and establishing oversight committees will ensure representation of the patient voice. Developing a safety incident repository and other relevant safety data can help patients to share their experiences with partners across the chiropractic profession.

#### Objective 5. Health worker education, skills, and safety

The *WHO Global Patient Safety Action Plan* fifth strategic objective addresses workforce development with attention to the necessary skills to sustain a patient safety culture. Various health professions have developed safety-focused educational competencies [26, 27]. However, professional training curricula have not fully incorporated interdisciplinary patient safety education alongside basic science and clinical knowledge development [28]. 

### Considerations

Chiropractic training programs can lead this movement by integrating patient safety curricula as an integral component of professional education. Developing an informed chiropractic workforce capacity with appropriate skills is likely to improve both familiarity and proficiency in addressing safety with patients and in responding to potential safety events.

### Call to action

The chiropractic profession must establish and teach comprehensive patient safety competencies across its training programs worldwide. Professional development and continuing education should explicitly incorporate patient safety training to emphasize the chiropractor’s role in minimizing harm and in coordinating with broader healthcare and public health systems. Innovative simulation methods to develop competence in patient safety are needed for all chiropractic education and training stakeholders. Patient safety centers of excellence that provide leadership on safety concepts, research, and education for the profession should be developed and supported with adequate resources.

#### Objective 6. Information, research, and risk management

The *WHO Global Patient Safety Action Plan* sixth strategic objective hinges on safety data. Data infrastructure, ecosystem, and culture promote system interoperability, event monitoring, information exchange, and research to reduce avoidable harm in healthcare. Digitalization of healthcare through widespread adoption of electronic health records and digital health tools streamlines data collection from a variety of sources for patient safety monitoring [29]. 

### Considerations

Chiropractic needs to collect, analyze, and publish high-quality, transparent safety data to function as a learning profession. Electronic systems and modern computational methods can support real-time risk management for anticipative and proactive learning and rapid adaptation, rather than reactive management, of patient safety events [29–31]. Ensuring that resource-challenged communities are meaningfully engaged in development is critical to maximize the adoption of a global network of health information systems. Community engagement can support resource planning, infrastructure development, and cultural adaptation of health information systems [32]. 

### Call to action

The chiropractic profession should develop transparent patient safety reporting information systems, with interdisciplinary input from healthcare personnel, clinical leaders, policymakers, informaticians, implementation scientists, patients, and resource-challenged communities within high, middle, and low-income countries. Standard vocabularies and common data models should build on foundational work in chiropractic [33] and other healthcare domains [34–36] given the cross-cutting nature of patient safety reporting systems.

#### Objective 7. Partners, synergy, and solidarity

The seventh objective of the *WHO Global Patient Safety Action Plan* calls for multinational and multisector partnerships to improve patient safety and healthcare quality. Collaboration and partnership are essential across a patient’s healthcare team, including chiropractors, to optimize patient safety in a holistic, patient-centered approach [37]. 

### Considerations

Central advocacy groups and leaders with strategic vision, such as the WFC Global Patient Safety Task Force, can guide patient safety initiatives, but collaboration across the profession is essential to implement and sustain these projects. A concise narrative that articulates current perspectives, visions, goals, and strategies for the chiropractic profession is important to synergize patient safety efforts alongside healthcare partners.

### Call to action

The chiropractic profession must promote and lead unified patient safety initiatives within the profession and across the broader healthcare environment. Chiropractic stakeholders in private and public sectors, including chiropractors, professional organizations, academic institutions, research centers, funders, insurers, industry, and, most importantly, patients and families, must match objectives of patient safety efforts and regularly convene meetings to innovate, implement, and sustain professional goals related to patient safety.

## Conclusion

Informed by the *WHO Global Patient Safety Action Plan*, this commentary is a pivotal call to action for the chiropractic profession to embrace and advance a comprehensive and committed patient safety culture. The World Federation of Chiropractic Global Patient Safety Task Force has established a framework for action by defining essential considerations and actionable steps aligned with the strategic objectives of WHO. This alignment allows for a cohesive approach to patient safety and lays the groundwork for future safety initiatives. Ongoing efforts by the WFC Global Patient Safety Task Force to advance patient safety include a scoping review of implementation strategies for patient safety culture within chiropractic and a systematic review of addressing patient safety culture in all healthcare settings broadly. By embracing a call to action guided by the *WHO Global Patient Safety Action Plan* framework, the aspiration is for the chiropractic profession to minimize preventable harm, proactively address risk and emerge as a leader in universally safe care for all individuals.

### Electronic supplementary material

Below is the link to the electronic supplementary material.

Supplementary Material 1

## Data Availability

Data sharing is not applicable as no datasets were generated or analyzed. Readers are encouraged to review the World Health Organization Global Patient Safety Action Plan 2021–2030 (who.int) for detailed recommendations and implementation strategies to support patient safety initiatives in their healthcare facilities.

## Abbreviations

GPS: Global Patient Safety
WFC: World Federation of Chiropractic
WHO: World Health Organization
