# Supplementary material for: The World Federation of Chiropractic Global Patient Safety Task Force: a call to action
Source: Chiropr Man Therap. 2024 May 13;32:15. doi: 10.1186/s12998-024-00536-1 (PMC11092090; doi:10.1186/s12998-024-00536-1)
Supplement: Supplementary file 1 — Supplementary Material 1 [file 12998_2024_536_MOESM1_ESM.docx]

**Supplemental Table 1.** Considerations and Call to Action Items of the WHO Global Patient Safety Action Plan framework in the context of the chiropractic profession.

| **Framework Strategic Objective** | **Considerations to Prioritize** | **Calls to Action** |
| --- | --- | --- |
| Objective 1. Policies to eliminate avoidable harm in health care | - Patient safety is a top priority across healthcare, including the chiropractic profession. - Patients and professions may thrive when minimum standards for patient safety are established. - Awareness of patient safety in all healthcare settings is an important first step. Collaboration with other health professions, patient groups, and academic and research centers globally is essential to promote, prioritize and immerse patient safety into the chiropractic profession. - Patient safety policies must move beyond the potential harms of diagnostic tools or therapeutic interventions to focus on informed consent, equitable care delivery, trusted communication, and more. | - Adopt policies that affirm a global and equitable commitment to patient safety as a health priority across the chiropractic profession. - Critically review policies and adopt professional standards to ensure patient safety is engrained in the chiropractic professional culture. - Ensure patient safety is a component of licensing, continuing education, regulatory, and accreditation requirements. - Budget sufficient funding and human resources to achieve patient safety action goals. - Establish a global research agenda and evidence base for patient safety in the chiropractic profession. - Observe World Patient Safety Day annually on 17 September to increase awareness worldwide. |
| Objective 2. High-reliability Systems | - As excellence in patient safety involves more than a focus on negative incidents, develop learning mechanisms to collect information on patient harms, near misses, and positive outcomes. - A just culture recognizes complexities in healthcare environments and that most safety incidents are attributable to systemic shortcomings (versus few that are from willful negligence or misconduct from an individual). - Recognize the difference between the Safety-I and Safety-II frameworks. | - Learn from high-reliability industries with respect to safety monitoring and response. - Commit to sustaining a resilient chiropractic profession based on both the Safety-I and Safety-II frameworks. - Conduct regular surveillance and critical appraisal to understand what creates success, to identify gaps, and to measure change within an organizations’ patient safety culture. - Ensure protection mechanisms are based on learning from patient safety failures and refining the work system, rather than punishing individuals. Ensure these are widely available and known to all stakeholders. - Define clear-cut boundaries and distinctions between errors and negligence to establish a just culture and facilitate appropriate corrective actions. - Establish strong patient safety leadership that puts expertise ahead of organizational hierarchizations to create resilient and just environments at all levels. |
| Objective 3. Safety of clinical Processes | - Safe clinical process designs must be tailored for diversity in clinical settings, including chiropractic clinical settings. - Communication and teamwork are key patient safety pillars in all clinical settings. - Clinicians are advocates for the inclusion of patient safety for all healthcare organizations they belong to. - Reporting adverse events from clinical processes is essential to maintain public trust. | - Optimize communication processes between chiropractors and the broader healthcare system. - Standardize assessment tools to identify patients at risk for potential safety events from chiropractic care. - Implement standard operating procedures, clinical pathways, health record documentation and reporting formats to assure consistent diagnosis, treatment, referral, and follow-up for chiropractic patients who experience safety events. Disseminate evidence-based resources that identify clinical processes to reduce the risk of patient safety incidents across chiropractic settings. - Identify risk-prone clinical procedures and effective mitigation strategies to reduce avoidable harm from chiropractic care. - De-implement and stop teaching clinical procedures that pose unfavorable risk/benefit safety profiles for chiropractic patients. - Commit to evidence-based processes for delivery of clinical care in clean and hygienic environments. |
| Objective 4. Patient and family Engagement | - Patient empowerment is a powerful and essential tool to improve patient safety, and yet is often missing from conversations about patient safety. - Patient and family advocates should be valued, supported, and listened to for cultures of safety and respect to thrive in chiropractic healthcare settings. - Patient and family engagement must be integral to healthcare practice, professional training, and patient safety initiatives. - Patients, families, and communities must have access to health records and information needed to manage their health and protect their own safety. - Chiropractic care settings must commit to policies of informed consent, full patient disclosure of any harm, and active patient safety reporting systems. | - Empower partners with patient-centered educational materials on the benefits and safety risks of chiropractic care. - Train on how to engage in constructive dialogue with patients. - Appoint patients and family members to chiropractic committees and organizations to advocate for patient safety initiatives and advise about avoidable harm. - Create community oversight and patient assistance programs to support people who encounter safety problems from chiropractic healthcare. - Organize patient/family story events and repositories to share experiences of avoidable harms and efforts to improve safe delivery of chiropractic healthcare. - Develop an international patient safety charter to promote the concept of safe chiropractic care. - Collect and analyze meaningful outcome measures of patient safety experiences in chiropractic healthcare to assess progress toward safety goals. |
| Objective 5. Health Worker education, skills, and safety | - Chiropractic training programs and continuing professional development do not sufficiently emphasize patient or health worker safety topics. - Formal training can help develop clinician comfort and competence in discussing safety with patients and addressing potential patient safety events. - Patient safety education is essential for all staff and patients involved in the chiropractic clinical setting. - Advanced training in patient safety frameworks and methods may be valuable for those in leadership roles. | - Review and revise chiropractic education competencies to embed issues of patient safety. - Develop patient safety training for stakeholders at all levels, including the use of simulation methods to train and develop chiropractor competence. - Incorporate patient safety within clinical education, including on-the-job and leadership training, for all members of the chiropractic care team. - Designate and support a patient safety center that provides leadership on patient safety concepts, research, and education for the profession. - Provide adequate resources for the sustainability of education and policy pertaining to health, safety, and environmental and occupational health. |
| Objective 6. Information, research, and risk management | - Collection and analysis of high-quality, veracious data pertaining to patient safety and the chiropractic care setting are foundational to functioning as a learning health profession. - Interoperability of the electronic information systems within chiropractic clinics is part of the larger healthcare ecosystem, and it has a multi-faceted effect on the overall safety of patients. - Advancing computational methods and data mining techniques (e.g., machine learning) may be useful for active surveillance of patient safety events. - Standardized communication practices pertaining to the domain of patient safety support patient safety capacity in all healthcare, including the chiropractic profession. | - Develop a patient safety reporting information system, with interdisciplinary input from clinical leaders, policy makers, informaticians, implementation scientists, and non-healthcare personnel. Conscious attention should be given to engaging all levels of chiropractic care stakeholders – including patients. Further, engaging with resource-challenged communities is paramount to ensure equitable participation and implementation globally. - Develop ontologies and knowledgebases, standard vocabularies, and/or common data models to support patient safety reporting systems relevant to the chiropractic profession. - Establish a research agenda for safe digital health and information technology integration within the chiropractic care setting. |
| Objective 7. Partners, synergy, and solidarity | - The chiropractic profession requires advocacy and prioritization of patient safety at a high level, with leaders having a strategic plan to guide implementation at all stakeholder levels. - Collaboration with diverse partners within and outside the chiropractic profession are critical to patient safety in the larger healthcare ecosystem. - It is critical for the chiropractic profession to have a concise narrative that articulates current perspectives, goals, and strategies. | - Reduce siloed efforts and promote a unified patient safety movement across the chiropractic profession and in efforts across healthcare more broadly. - Establish networks of experts and representatives in all sectors (i.e., professional organizations, academic institutions, research centers, patient/private sectors, and industry) for collaboration in support of patient safety initiatives. - Match goals and objectives of global plans to respective stakeholder plans in patient safety efforts. - Regularly convene meetings to innovate, implement, and sustain professional goals related to patient safety. |
